# Round the Hospitals

**Published:** 1921-03-26

**Authors:** 


### ROUND THE HOSPITALS.

It will come upon nurses with something of a shock to learn that the final decision of the Minister of Labour in regard to nurses and probationer nurses in voluntary hospitals is such as to make them "employed persons within the meaning" of the Unemployment Insurance Act. The question of whether sisters in voluntary hospitals also come within the scope of the Act is still under consideration. The immediate effects of this decision will make themselves felt in the form of a tax on the nurses and on the institutions they serve. The present contribution is 6½d., but after July the nurse must pay 4d. a week, about 17s. a year, for her part. The institution must pay 5d., about £1 1s. a year, for each nurse. In neither case will payment of such contributions be very welcome. The practical unanimity of the profession against inclusion in the Unemployment Insurance Act was due to the failure of the promoters of the Act to show how it could benefit this particular class of workers. No probationer is ever out of work unless she be unsuited by mind, morals, or bodily capacity for this occupation. The same may be said of institution nurses. It is clearly a measure designed

**ROUND THE HOSPITALS—(*continued*).**

for some possible future contingency rather than for the present, a class of legislation which must always be regarded with suspicion. When it has been clearly demonstrated by the working of the Act for a year or two that institution nurses and probationers cannot in the nature of things expect to receive a fair return in benefits from their contributions, the profession will have a good case for the repeal of the clause which has drawn them against their will within the meshes of the scheme.

In consequence of representations by the Minister of Pensions that it is desirable to make further provision concerning the pensions and grants sanctioned by the Order in Council of September 29, 1917, for members of His Majesty's Naval Forces, including the Royal Naval Nursing Service, disabled in consequence of the Great War, new rates of pensions, allowances and gratuities have been sanctioned. These are to come into force as from April 1, 1920, and are in substitution for those of September 29, 1917, provided that if more favourable to the nurse the pension allowance or gratuity already payable shall be continued instead of the newly sanctioned scale, and that no grant shall be reassessed to the disadvantage of the recipient. The new scale was published in full in the *London Gazette* of March 18, and nurses of the Royal Naval Nursing Service to whom it may apply should communicate with the Secretary, the Ministry of Pensions.

The Ministry of Pensions desires to make it as widely known as possible that every effort is being made to ensure that nurses who have served in the late war and have become disabled by reason of such service shall be substantially aided. Members of the regular Q.A.R.N.N.S., of Q.A.I.M.N.S., of the Reserves, of the R.A.F.N.S., and of the T.F.N.S., as well as other nurses who have been employed regularly and full time on nursing Naval, Imperial, Military or Air Force *personnel* in hospitals or under recognised organisations, are included in a well-thought-out scheme of benefits, and if eligible for pensions or gratuity should make application to the Secretary, Ministry of Pensions, Cromwell House Annexe, Millbank, S.W. 1. Applications for training for another employment, if the war service has rendered the nurse unfit for her career, should be made to the Ministry of Labour, St. Ermin's, Caxton Street, S.W. 1. There is a Special Grants Committee at Millbank House, S.W. 1, which supplements pensions and sometimes makes grants to the dependants of disabled nurses. There are conditions under which pensions or gratuities may be granted to the relatives of nurses who have lost their lives in the war. And there are funds in existence to assist cases of distress not covered by the above. The right course for a nurse in doubt or difficulty is to write to "The Officer's Friend," Ministry of Pensions, Cromwell House, Millbank, S.W. 1, when she will learn exactly what can be done for her.

Lady Astor has been interesting herself in the pay of Army nurses, and on March 13 she asked the Secretary of State for War whether he was aware of the poor pay of members of Q.A.I.M.N.S. —£60 to £65 for staff nurses, £75 to £85 for sisters—and whether he will consider the desirability of revising this scale so as to attract the right type of woman to this branch of the Service. The reply was exceedingly discouraging. But it is much for the good of the nursing services that our woman member should realise and make known to the House of Commons the inferiority of nurses' pay as contrasted with that of other servants of the State, and nurses will no doubt accord her their gratitude.

The Workhouse Nursing Association has addressed a valuable letter to Boards of Guardians pressing the needs of those patients unable to obtain nursing accommodation in hospitals, and asking for their favourable consideration. The Society points out that its attention has been called to the needs of a large class of persons whose financial position, whilst formerly such as enabled them to maintain their independence, is now so impaired by the war that in sickness or infirmity their condition is one of real privation. In view of the distress to which many of them are reduced the Society considers it to be most desirable that such persons, especially chronic or incurable cases which from their nature preclude them from treatment in general hospitals or nursing homes, should be able to take advantage of the admirable provision afforded by Poor-law infirmaries. Accordingly a deputation of their members approached Dr. Addison, the Minister of Health, and in his reply Dr. Addison expressed himself as being in the fullest sympathy with the proposition and with the class of persons on whose behalf they were pleading. He pointed out, however, that the whole Poor-law administration is being reviewed, and that, in his opinion, the sick ought not to be under the control of the Guardians; but until any change is effected the matter in question would be best dealt with by the authorities concerned. He added that the question of classification of the patients is a difficult one, but the payment proposed may very fairly secure the advantage of treatment in separate wards. As some time might elapse before the general law is altered, and as the need for treating these cases is very urgent, the Society asks the Boards of Guardians whether it would be possible for them to meet this crying need by allocating wards to such cases as described and providing for them in a manner suited to their requirements. The burden upon the rates would not be heavy, as in the majority of cases a considerable proportion of the cost could be recovered from patients.

The number of female nurses from mental hospitals in all parts of England and Wales who succeeded in passing the examination of the Medico-Psychological Association in November last and obtaining a certificate was exactly one hundred. What are the mental hospitals about? Their staffs number many thousands. Taking the asylums one with another, they should each be training, on an average, fifty probationers, and sending up at least ten candidates a year for the final examinations. Yet out of the whole total of asylums and mental institutions in this country we have a pass list of one hundred. It is pretty certain that this marks a serious falling-off in the candidates prepared for these examinations. Referring to Burdett's "How to Become a Nurse," we find on page 317, under the heading of "Training Schools for Attendants on the Insane," that "over 1,000 candidates present themselves annually for examination by the Medico-Psychological Association. The inquiries we have made lead us to conclude that there are very exceptional cases where attendants are not systematically trained." Can this be said at the present time?

---

Thomas Edward Wilcock, aged thirty-one, the male nurse to whose prosecution for assault on his patient, the Rev. Canon William Banham, aged eighty-eight, we recently referred, was convicted at Leeds on March 14 of manslaughter and sentenced to eighteen months' hard labour. At the inquest on the Canon medical evidence showed that death was due to exhaustion following injuries to the mouth, neck, and throat. Such cases are extremely rare, and for that reason are all the more horrifying.

